# The Relationships between Internal and External Load Measures for Division I College Football Practice

**DOI:** 10.3390/sports8120165

**Published:** 2020-12-15

**Authors:** Eric J. Sobolewski

**Affiliations:** Molnar Human Performance Lab, Department of Health Sciences, Furman University, Greenville, SC 29613, USA; eric.sobolewski@furman.edu; Tel.: +1-864-294-3602

**Keywords:** speed, distance, heart rate, TRIMP, RPE

## Abstract

The aim of this study is to explore the relationships between internal and external load measures in American football. Thirty football players wore a portable integrated monitor unit for 10 weeks during the fall football season. Relationships between internal and external load measurements were determined. Internal load consisted of heart rate zones and heart rate-derived measures and session Ratings of Perceived Exertion (sRPE). External load consisted of distance in different speed zones, total distance traveled, and accelerations. There were many significant positive relationships, but the meaningful relationships (*r* > 0.5) were between heart rate-derived measures of load (Training Impulse and heart rate reserve) and low-intensity movement and total distance. Only accelerations between 1 and 1.99 m·s^−2^ were moderately correlated to heart rate-derived internal load. RPE values alone did not correlate strong enough with any of the measure but sRPE training load (sRPE-TL) correlated to most external values. Overall, moderate correlations were present between heart rate-derived internal load to total distance and lower intensity movement. sRPE-TL values had high correlations but were highly dependent on duration, not perceived exertion. When addressing load in American football, duration of the session is a key component in determining internal load as HR data and sRPE alone do not correlate highly with external loads.

## 1. Introduction

In recent years, the development of technology has allowed researchers and practitioners to get a better understanding of the loads placed on athletes, both during competition and practice [1]. These training loads are commonly used to determine if enough training stimulus is prescribed to the athletes to cause positive adaptation [2], but also if too much load is given which could result in fatigue and injury [3]. There is evidence that monitoring load does have practical application to sport performance [3] and thus has become an integral part of an athlete management system. The three most common ways to measure load are from Global Positioning Systems (GPS), session Ratings of Perceived Exertion (sRPE), and Heart Rate (HR) Monitors. External load is commonly measured from GPS units that are worn by athletes. GPS very often contains a built in accelerometer. From these devices, training loads [4,5] are calculated and other metrics such as speed zones, accelerations, and impacts [5,6,7,8,9] are often reported. For internal load, sRPE is a common measure simply reported by the athlete [10] and has been used to extrapolate training load by multiplying sRPE and the duration of the training session (sRPE-TL). HR data is also a common metric, which allows for the analysis of the heart rate response to training. HR data can be broken down into percentages, or more commonly reported as load in the form of a training impulse (TRIMP) value [5,6]. The use of one or all of these metrics have been reported in all major field-based team sports: soccer [11], rugby [12], Australian Rules football [7], and American football [6,13,14].

The evaluation of training loads is sport- and team-dependent as each sport and team have their own unique style of play and practice [3]. Understanding of what makes up these loads is vital to understanding the stresses placed on and by the human body during sport. External loads are associated with physical work being done by the body in the form of movement, while internal loads are measures of a combination of biochemical and the biomechanical stress on the system [2]. The stress placed on human body varies drastically between sports as some sports are highly linear and aerobic in nature, which lends to using the measures of heart rate and distance to account for load (cycling, running). Other sports are more variable in nature, having bouts of high-intensity sprints followed by periods of aerobic recovery (rugby, football, and soccer). The duration and intensity of these periods vary significantly between and within sports (different practice types) and differ between positions on the field [2,13]. In a meta-analysis evaluating the magnitude of the relationships between internal and external load in team sports, McLean et al. [2] concluded that “magnitudes are dependent on the specific measures used and are substantially moderated by training mode.” In their analysis, they looked at multiple sports and found common measures of TRIMP, sRPE, and sRPE-TL for internal loads and total distance, high-speed running distance, very high-speed running distance, accelerometer load, and impacts. The highest relationship came from total distance and accelerometer load data to both sRPE-TL and TRIMP values, with the Pearson’s product coefficients ranging from 0.51 to 0.79. It is worth noting that not a single research study on American Football was included in the meta-analysis. Since every sport has unique demands placed on the players, it would be of interest to evaluate these relationships in American Football.

To date, there is limited research that evaluates a measure of internal load with an external load in American football. Govus et al. [10] reported a trivial relationship of sRPE-TL and accelerometer-derived load. Flatt et al. [15] addressed internal load using heart rate variability and acceleration-derived load, but was used to evaluate chronic workload and recovery, however they did not address individual load of practice. One reason for limited data on American football is that load is typically measured via GPS units placed on the back of athletes (via strap or in shoulder pads) and they either do not have HR measuring capabilities or compatible HR monitors were not worn. Very few studies [6,13] have both internal and external measure of load on American football players but have not addressed the relationships of internal and external loads. With the demands of American football being vastly different from other field-based sports and the lack of research in American football, it would be highly important to determine the relationship between internal and external loads in this sport. Therefore, the aim of this study is to explore the relationships between internal and external loads measures in American football.

## 2. Materials and Methods

### 2.1. Participants

GPS, HR, and sRPE were collected during normal practices of a Division I college football bowl subdivision team over a 10-week period (from August through October). There was a potential for 1020 observations, but due to incomplete data from either unit not being worn, times where GPS or HR lost signal, and no matching data for HR, GPS, and sRPE, there were 550 comparable observations. Data was collected as part of a monitoring program in place to determine the wellbeing of the student athlete. Data was gathered from 30 football players (age, 20.0 ± 1.0 years; mass 101.8 ± 21.5 kg; height 183.4 ± 9.2 cm). All data and information met the University’s Intuitional Review board’s approval (IRB #2016-246). All data presented was calculated from GPS, accelerometer, HR, and sRPE data. Data was collected on padded practices (helmets and shoulder pads) which varied from week to week of either three or four practices, depending on schedule. Friday walk through and Monday recovery days were not evaluated in this study.

### 2.2. Procedure

Monitoring devices: GPS and HR data were taken together using a commercially available unit (Polar team Pro: Polar, Polar Electro Oy, Kempele, Finland) which had a sampling frequency for the GPS at 10 Hz, accelerometer at 200 Hz, and 1000 Hz for the HR sensor. The Monitoring device (39 g, 36 × 68 × 13 mm) was placed around the chest with the sensor position across the xiphoid process. This monitor for HR has been shown to be a valid and reliable measure of HR [16]. GPS has been proven to be both reliable and valid in measuring distance and velocity during high-intensity exercise [17], while acceleration data has been proven to be reliable when reporting bands of acceleration rather than absolute values [18]. All GPS, HR, and accelerometer data collected from the devices were filtered and presented by Polar proprietary algorithms.

### 2.3. Session Ratings of Perceived Exertion

Session Ratings of Perceived Exertion (sRPE) was collected following practice on a mobile App. The App asked a series of questions, one of which asked the players to rate the practice on a modified Borg’s CR10 scale [19] from 1 to 10, with 10 being the hardest/max effort practice and 1 being light/hardly any effort. Players were familiar with this scale as it had been implemented during the summer conditioning program. sRPE-training load (sRPE-TL) was simply the sRPE multiplied by total duration time of the training session in minutes [10].

### 2.4. Heart Rate Data

The following HR zones have been used in previous monitoring literature [5,13] and were used for analysis: 0–60%, 60–70%, 70–80%, 80–90%, and 90–95%. Heart rate zones where determined from a progressive test in which max HR was recorded. The test was a modified multiple stage fitness test in which distance remained constant and rest time and goal times decreased. Different position groups ran different distances (15–35 m) with different rest and goal times, as this was a sport- and position-specific test. Total HR exertion was calculated using Edwards internal training load method [20]. TRIMP [19] was calculated as:

$$\mathrm{TRIMP}\;=\;TD\times HR_{R}\times 0.64e^{1.92\;\times \;HR_{R}}$$

The percent HR reserve (% HR reserve) was calculated for each practice using the formula developed by Karvonen [21].

All other HR data (Max, Average, HR load, Energy Expenditure, and Recovery) values were calculated by proprietary algorithms that where provided by Polar [13].

### 2.5. External Loads

The following distances traveled in each speed zone were used for analysis, as used in previous research [13]: Standing and Walking (0–6 km·h^−1^), Jogging (6–12 km·h^−1^), Cruising (12–14 km·h^−1^), Striding (14–18 km·h^−1^), High-Intensity Running (18–20 km·h^−1^), and Sprinting (>20 km·h^−1^). Data was also divided into Low-Intensity Distance (0–14 km·h^−1^) and High-Intensity Distance (>14 km·h^−1^). High- and Low-Intensity Durations were then used to formulate a Work to Rest ratio [5]. From GPS data, the following averages were recorded: Max speed, Number of Sprints (bursts over 20 km·h^−1^), and Total Distance.

Acceleration data: Accelerations were broken down into deceleration and acceleration efforts. Each effort was classified into one of four categories: 0.5–0.99, 1–1.99, 2–2.99, and >3 m·s^−2^.

### 2.6. Statistical Analyses

Pearson’s product correlations were run between each measure of internal load and external load. Within-athlete comparisons have used Pearson’s *r* [2] and are an appropriate method for analyzing repeated measures data [22]. Tests for normality were conducted using Shapiro–Wilk’s tests. All statistical analyses were performed using Statistical Package for Social Science (IBM SPSS Statistics for Windows, Version 23.0. Armonk, NY, USA). Alpha was set at *p* = 0.05. An *r* value (>0.5) of a moderate correlation was seen as a stronger and more informative relationship than just significance alone [23]. Results of the Shapiro–Wilk test concluded that the majority of the data was normally distributed with a few variables that were not normally distributed (*p* < 0.05): maximal speed, maximal and minimal heart rate, and polar recovery.

## 3. Results

All Pearson’s product correlations are presented in Table 1, Table 2 and Table 3. There were many significant relationships, however many had low *r* values and provided little insight into the internal and external relationship.

## 4. Discussion

Understanding the relationships between internal and external loads in American football are important in gaining insight into the demands placed on the athletes. One of the main findings of this study was that heart rate data alone did not have any substantial relationships to external load, it was only when metrics were derived from HR data that meaningful correlations were present. HR load, total HR exertion, TRIMP, and estimated energy expenditure are all derived from HR intensity and duration of training session. These measurements had the highest correlation coefficients of any of the HR-derived internal load measurements. Another main finding is that for sRPE relationships, there were significant relationships, but they were minimal at best. The highest relationships of all internal load measures were for sRPE-TL, as correlations varied from moderate to high in most external variables (0.60–0.92). The difference between sRPE and sRPE-TL was the duration of practice. Longer practices would result in more opportunity to experience more external load. This is seen as the average sRPE was 7.4 ± 0.3, indicating that there was little variation in the sRPE of practice, indicating that duration played a larger role in load then sRPE when comparing to external loads. As many of the load relationships yielded significant relationships, only a few had moderate to strong correlations. Taking into consideration both sRPE-TL- and HR-derived measures of internal load, it appears that duration is a key indicator when analyzing internal load.

Further diving into the HR-related measures of internal load, the highest relationship values were only present in the lower intensity external load values, with high correlations of internal load to jogging and low-intensity distance values. At higher speed (>14 km/h), relationships were significant but not meaningful. The largest relationships were observed with HR-derived load measures and total distance (*r* > 0.55) and low-intensity distance (*r* > 0.51). The high relationships of internal loads and to total distance is in line with previous literature that showed sRPE values highly correlating to total distance [2,24]. Bartlett et al. [25] put into context that sRPE was heavily reliant on total distance, as they contributed 87% of the sRPE score to total distance. As total distance appears to have the highest relationship to internal loads, many factors go into sRPE values, such as mental fatigue, position, playing experience, and fitness levels [25]. Therefore, HR-derived data might be a better indicator of internal load, while sRPE values might be a perception of load value. HR data and sRPE are highly correlated (0.5–0.6) [19], and have been suggested to be somewhat interchangeable, but as with all training loads, they are unique to the individual [24]. In response to this, we analyzed sRPE to HR-derived load measures with Pearson’s *r* values ranging from 0.22 to 0.26, however when we accounted for duration and analyzed sRPE-TL, the correlation coefficient ranged from 0.62 to 0.89.

This study and others [2,24] have demonstrated that the HR-based measures of internal loads do not correlate to high-speed running. This is of great importance as high-speed running places extreme biomechanical loads on the body [26]. This can be partially accounted for in acceleration data, but there is still a large gap in internal loads and external loads when addressing high-speed movement. The lack of relationships of high-speed running has been attributed to GPS error at high speeds [27], individual ability/requirement to reach high speeds [28], and the non-linear relationship between speeds and internal intensity [2]. One aspect that internal loads do not account for is higher speed movement (>14 km·h^−1^) and high accelerations (>2 m·s^−2^). These external variables are commonly understood as the most biomechanically demanding, yet based on our data, are not accounted for with internal load measures.

The demands of American football are different from other field-based sports, however this study demonstrated that internal loads are related to external loads in a similar fashion. According to McLaren et al. [2], football practice would be classified as a mix model, as normally, practice includes skill, neuromuscular, and metabolic efforts. Previous work [13,29] has demonstrated that the majority of football would be more sprint interval-based, with periods of high speed/intensity followed by longer periods of rest. Sobolewski et al. [13] reported work-to-rest ratios being greater than 1:16. The typical practice analyzed in this study had 5–7 periods, with each period lasting 15–30 min and having a specific purpose. Practice consisted of individual skills, special teams, small group games, and larger full-team periods. One of the biggest differences of American football to other sports is the unique position groups, as these periods were not all the same for each position group. For example, in offensive and defensive line individual skill periods, the players may be stationary and just be working on hand placement, while receivers maybe running routes of varying lengths. Overall, these groups have very different physical demands [6,13,29,30] and require different monitoring approaches. Even though previous literature [13] demonstrated that positions have similar total practice internal load values, team drills and position drills elicit different HR responses [6]. For example, even in a common team period, an offensive lineman on average travels five meters per play, while wide receivers may travel 20 or more meters per play. This would significantly change both total distance and HR values based off the positional demands, but sRPE values might remain similar, as they both interpret the loads according to their own specific perceived demand. It has been suggested [6] that in American football, practice demands should not be analyzed as a whole, but further broken down into periods or segments of practice to give a better indicator of intensity. Further research into American football needs to address differences in periods and positions on internal and external load variables. One common thread is that sRPE-TL and total distance in American football and other mixed model sports [2] had high correlation values.

A limitation is that the current software and hardware units used in this study do not calculate an external load from acceleration data, as previous literature that has demonstrated internal load relationships with acceleration-derived load values has done [2]. Another limitation is the use of HR data to evaluate HR zones, recovery, and energy expenditure, as the formulas used are extrapolated from aerobic-based performances and might not be applicable to mixed model performances. HR-derived internal measures where used as a possible variable that might have a significant relationship. The results demonstrated, as one would expect, that HR zones are not great predictors of sports that are not purely aerobic in nature. An energy system-based model might be hard to evaluate as American football has many demands that are position-specific, and practice is more of a mixed model with high anaerobic burst followed by long periods of rest. This type of model does not lend itself easily to HR measures of intensity.

Overall, internal load metrics have moderate to high correlations to external loads, with HR-derived and sRPE-TL demonstrating more consistent relationships, this, however, seems to be more dependent on duration rather than just intensity alone. Measures of internal and external load may be highly correlated but should not be used interchangeably to determine exercise prescription, periodization, and athlete management. Each load metric is unique to the measurement and practitioners need to determine what are the best metrics for analysis. Along with previous research [2,10,24,30], this study concluded that using one metric alone is not an effective approach when evaluating load in sport, as some metrics might not be applicable to mixed model sports like American football. This research and others are attempting to process out what is useful and non-useful data to allow practitioners to streamline their approach so there are not redundancies and over-analysis of load metrics.

## 5. Conclusions

This is the first study to address the relationship between internal and external measure of load in American football. The results suggest that duration of the training session is key for relationships between internal and external load measures. For measures that were HR-based alone, there were minimal relationships, indicating that HR may not be an effective way to gauge load in American football as the stop and go nature of the sport and practice does not yield itself to HR zone monitoring. In conclusion, even though relationships between internal and external loads exist, not a single value is comparable to another and each serve a unique purpose in understanding load management. When monitoring load in American football, duration of the training session should be strongly considered as a main determining factor when trying to account for load.

## Figures and Tables

**Table 1:** Relationships between internal load and speed zones (Pearson’s product correlation coefficients: *r*).

	Standing/Walking	Jogging	Cruising	Striding	High-Intensity Running	Sprinting
Polar HR load (a.u)	0.46 *	0.66 *	0.44 *	0.39 *	0.33 *	0.30 *
Zone 1: 0–60% HR max	0.47 *	0.24 *	0.16 *	0.21 *	0.19 *	0.20 *
Zone 2: 60–70% HR max	0.36 *	0.52 *	0.28 *	0.21 *	0.16 *	0.10 *
Zone 3: 70–80% HR max	0.26 *	0.51 *	0.32 *	0.26 *	0.23 *	0.17 *
Zone 4: 80–90% HR max	0.02	0.33 *	0.13 *	0.09 *	0.07	0.08
Zone 5: 90–95% HR max	0.01	0.07	0.06	0.04	0.06	0.16 *
Total HR Exertion	0.62 *	0.72 *	0.41 *	0.36 *	0.29 *	0.25 *
Average HR (bpm)	0.15 *	0.47 *	0.22 *	0.13 *	0.05	0.01
Max HR (bpm)	0.06	0.16 *	0.15 *	0.10 *	0.12 *	0.06
Min HR(bpm)	0.03	0.05	0.04	0.03	−0.02	−0.02
% HR Reserve (a.u.)	0.10 *	0.33 *	0.10 *	0.03	0.01	−0.01
TRIMP (a.u.)	0.42 *	0.60 *	0.26 *	0.24 *	0.16 *	0.14 *
eEE (kcals)	0.54 *	0.73 *	0.40 *	0.31 *	0.23 *	0.18 *
Polar Recovery	0.13 *	0.48 *	0.20 *	0.08 *	0.02	−0.03
sRPE (borg 0–10)	−0.20 *	−0.17 *	−0.14 *	−0.15 *	−0.13 *	−0.02
sRPE-TL (a.u.)	0.92 *	0.85 *	0.71 *	0.65 *	0.67 *	0.71 *

* denote significant *r* values (*p* < 0.05), **Bold** indicates moderate to strong Correlation (*r* > 0.5). Time spent in each HR zone (min), Distance travel in speed zones: Standing and Walking (0–6 km·h^−1^), Jogging (6–12 km·h^−1^), Cruising (12–14 km·h^−1^), Striding (14–18 km·h^−1^), High-intensity running (18–20 km·h^−1^), Sprinting (>20 km·h^−1^), eEE = Estimated Energy Expenditure, a.u. = arbitrary units, Srpe = session rating of perceived exertion, sRPE-TL = session rating of perceived exertion training load, bpm = beats per min.

**Table 2:** Relationships between internal and external measures of load (Pearson’s product correlation coefficients: *r*).

	Total Distance	Work to Rest Ratio	Low-Intensity Distance	High-Intensity Distance	Max Speed	# of Sprints	Average Speed
Polar HR load (a.u)	0.66 *	−0.34 *	0.66 *	0.38 *	0.21 *	0.31 *	0.11 *
Zone 1: 0–60% HR max	0.40 *	0.26 *	0.41 *	0.22 *	0.13 *	0.20 *	−0.10 *
Zone 2: 60–70% HR max	0.48 *	−0.18 *	0.51 *	0.18 *	0.05	0.12 *	0.04
Zone 3: 70–80% HR max	0.45 *	−0.28 *	0.45 *	0.24 *	0.06	0.18 *	0.12 *
Zone 4: 80–90% HR max	0.19 *	−0.15 *	0.20 *	0.09 *	0.03	0.08	0.14 *
Zone 5: 90–95% HR max	0.07	−0.01	0.05	0.08 *	0.11 *	0.15 *	0.11 *
Total HR Exertion	0.74 *	−0.08	0.77 *	0.34 *	0.13 *	0.27 *	0.01
Average HR (bpm)	0.32 *	−0.40 *	0.35 *	0.08	−0.04	0.01	0.09 *
Max HR (bpm)	0.14 *	−0.09 *	0.14 *	0.11 *	0.01	0.06	0.04
Min HR(bpm)	0.04	−0.08	0.05	0.01	−0.03	−0.02	−0.03
% HR Reserve (a.u.)	0.21 *	−0.29 *	0.24 *	0.02	−0.02	0.01	0.12 *
TRIMP (a.u.)	0.55 *	−0.18 *	0.58 *	0.19 *	0.07	0.15 *	0.09 *
eEE (kcals)	0.69 *	−0.19 *	0.73 *	0.27 *	0.08	0.19 *	0.02
Polar Recovery	0.31 *	−0.16 *	0.35 *	0.04	−0.04	−0.02	0.05
sRPE (borg 0–10)	−0.21 *	−0.22 *	−0.18 *	−0.11 *	0.11 *	−0.02	0.04
sRPE-TL (a.u.)	0.89 *	0.18 *	0.91 *	0.69 *	0.45 *	0.72 *	0.22 *

* denote significant *r* values (*p* < 0.05), **Bold** indicates moderate to strong Correlation (*r* > 0.5). Time spent in each HR zone (min), Distance (m), Work to Rest Ratio (a.u.), High-intensity running (18–20 km·h^−1^), Sprinting (>20 km·h^−1^), Low Intensity Distance: 0–14 km·h^−1^ (m), High Intensity Distance: >14 km·h^−1^ (m), # of Sprints (>20 km·h^−1^), Speed (km·h^−1^), eEE = Estimated Energy Expenditure, a.u. = arbitrary units, sRPE= session rating of perceived exertion, sRPE-TL = session rating of perceived exertion training load, bpm = beats per min.

**Table 3:** Relationships between internal loads and accelerations (Pearson’s product correlation coefficients: *r*).

	Decelerations (m·s^−2^)				Accelerations (m·s^−2^)			
	>–3	2.99–2	1.99–1	0.99–0.5	0.05–0.99	1–1.99	2–2.99	>3
Polar HR load (a.u)	0.34 *	0.34 *	0.44 *	0.45 *	0.39 *	0.54 *	0.46 *	0.36 *
Zone 1: 0–60% HR max	0.18 *	0.16 *	0.13 *	0.11 *	0.19 *	0.19 *	0.17 *	0.02
Zone 2: 60–70% HR max	0.12 *	0.21 *	0.31 *	0.23 *	0.27 *	0.33 *	0.17 *	−0.01
Zone 3: 70–80% HR max	0.20 *	0.32 *	0.30 *	0.25 *	0.19 *	0.33 *	0.31 *	0.26 *
Zone 4: 80–90% HR max	0.19 *	0.18 *	0.25 *	0.24 *	0.23 *	0.26 *	0.29 *	0.25 *
Zone 5: 90–95% HR max	0.08	0.09	0.18 *	0.14 *	0.13 *	0.11 *	0.22 *	0.15 *
Total HR Exertion	0.23 *	0.35 *	0.47 *	0.37 *	0.39 *	0.49 *	0.37 *	0.16 *
Average HR (bpm)	0.28 *	0.40 *	0.51 *	0.43 *	0.42 *	0.54 *	0.44 *	0.26 *
Max HR (bpm)	0.17 *	0.19 *	0.19 *	0.22 *	0.16 *	0.22 *	0.26 *	0.29 *
Min HR(bpm)	0.05	0.03	0.04	0.07	−0.04	0.03	0.10 *	0.19 *
% HR Reserve (a.u.)	0.06	0.03	0.06	0.11 *	0.08	0.11 *	0.08	0.04
TRIMP (a.u.)	0.11 *	0.16 *	0.14 *	0.11 *	0.14 *	0.14 *	0.16 *	0.14 *
eEE (kcals)	0.22 *	0.34 *	0.37 *	0.29 *	0.33 *	0.39 *	0.32 *	0.20 *
Polar Recovery	0.28 *	0.38 *	0.47 *	0.42 *	0.39 *	0.50 *	0.43 *	0.30 *
sRPE (borg 0–10)	−0.15 *	−0.02	−0.11 *	−0.08	−0.15 *	−0.12 *	−0.13 *	−0.08
sRPE-TL (a.u.)	0.65 *	0.76 *	0.77 *	0.78 *	0.74 *	0.71 *	0.73 *	0.60 *

* denote significant *r* values (*p* < 0.05), **Bold** indicates moderate to strong Correlation (*r* > 0.5). Time spent in each HR zone (min), eEE = Estimated Energy Expenditure, a.u. = arbitrary units, sRPE = session rating of perceived exertion, sRPE-TL = session rating of perceived exertion training load, bpm = beats per min.
